# Two mechanisms of the enhanced antibody-dependent cellular cytotoxicity (ADCC) efficacy of non-fucosylated therapeutic antibodies in human blood

**DOI:** 10.1186/1471-2407-9-58

**Published:** 2009-02-18

**Authors:** Shigeru Iida, Reiko Kuni-Kamochi, Katsuhiro Mori, Hirofumi Misaka, Miho Inoue, Akira Okazaki, Kenya Shitara, Mitsuo Satoh

**Affiliations:** 1 Tokyo Research Laboratories, Kyowa Hakko Kogyo Co, Ltd, 3-6-6 Asahi-machi, Machida-shi, Tokyo 194-8533, Japan

## Abstract

**Background:**

Antibody-dependent cellular cytotoxicity (ADCC) has recently been identified as one of the critical mechanisms underlying the clinical efficacy of therapeutic antibodies, especially anticancer antibodies. Therapeutic antibodies fully lacking the core fucose of the Fc oligosaccharides have been found to exhibit much higher ADCC in humans than their fucosylated counterparts. However, data which show how fully non-fucosylated antibodies achieve such a high ADCC in human whole blood have not yet been disclosed. The precise mechanisms responsible for the high ADCC mediated by fully non-fucosylated therapeutic antibodies, even in the presence of human plasma, should be explained based on direct evidence of non-fucosylated antibody action in human blood.

**Methods:**

Using a human *ex vivo *B-cell depletion assay with non-fucosylated and fucosylated anti-CD20 IgG1s rituximab, we monitored the binding of the therapeutic agents both to antigens on target cells (target side interaction) and to leukocyte receptors (FcγR) on effector cells (effector side interaction), comparing the intensities of ADCC in human blood.

**Results:**

In the target side interaction, down-modulation of CD20 on B cells mediated by anti-CD20 was not observed. Simple competition for binding to the antigens on target B cells between fucosylated and non-fucosylated anti-CD20s was detected in human blood to cause inhibition of the enhanced ADCC of non-fucosylated anti-CD20 by fucosylated anti-CD20. In the effector side interaction, non-fucosylated anti-CD20 showed sufficiently high FcγRIIIa binding activity to overcome competition from plasma IgG for binding to FcγRIIIa on natural killer (NK) cells, whereas the binding of fucosylated anti-CD20 to FcγRIIIa was almost abolished in the presence of human plasma and failed to recruit NK cells effectively. The core fucosylation levels of individual serum IgG1 from healthy donors was found to be so slightly different that it did not affect the inhibitory effect on the ADCC of fucosylated anti-CD20.

**Conclusion:**

Our results demonstrate that removal of fucosylated antibody ingredients from antibody therapeutics elicits high ADCC in human blood by two mechanisms: namely, by evading the inhibitory effects both of plasma IgG on FcγRIIIa binding (effector side interaction) and of fucosylated antibodies on antigen binding (target side interaction).

## Background

The clinical successes of therapeutic antibodies have been demonstrated by improvement of overall survival and time to disease progression in various types of malignant diseases, such as breast, colon, and hematological cancer [1-5]. As molecular-targeted therapeutics, therapeutic antibodies that bind to specific cell-surface antigens on target cells can induce cytotoxicity via the effector functions of antibody-dependent cellular cytotoxicity (ADCC) and complement-dependent cytotoxicity (CDC) through the constant region of the antibody (Fc), and apoptosis of target cells directly [6-9]. Recently, some clinical evidence based on genetic analysis of leukocyte receptor (FcγR) polymorphisms of cancer patients treated with anti-CD20 IgG1 rituximab and anti-HER2 IgG1 trastuzumab therapies has revealed that ADCC is one of the critical mechanisms responsible for the clinical efficacy of these therapeutic antibodies [10-14]. A significant correlation has also been reported between the clinical responses to the therapies and the low-affinity FcγR polymorphisms in rituximab-treated patients of systemic lupus erythematosus (SLE) and Waldenstrom's macroglobulinemia, in Crohn's disease patients treated with anti-TNF-α IgG1 infliximab, and in pregnant women with fetal hemolytic disease treated with anti-RhD [12,15-17]. Hence, ADCC enhancement technology is expected to play a key role in the development of therapeutic antibodies with improved clinical efficacy. Indeed, a number of preclinical and clinical trials using such non-fucosylated antibody therapeutics are now underway.

Although various therapeutic antibodies confer great benefits to patients, they also present serious issues of cost-effective performance; as it has been pointed out, some of the currently approved therapeutic antibodies are unable to induce more than remission of cancer despite the fairly high cost of the therapy. Recently, the inhibition of therapeutic-antibody ADCC by plasma IgG, probably through competition for binding of the therapeutics to FcγR on effector cells, is found to be at least partly responsible for the large dose requirement in antibody therapies [18,19]. On the other hand, fully non-fucosylated anti-CD20 rituximab is shown to exhibit strong ADCC at lower concentrations (10 to 100 ng/mL) with much higher efficacy than that of its fucosylated counterparts, even in the presence of plasma IgG [20,21]. However, the basic data elucidating how fully non-fucosylated antibodies show such a high ADCC in human whole blood have not yet been disclosed. The precise mechanisms responsible for the high ADCC mediated by fully non-fucosylated therapeutic antibodies, even in the presence of human plasma, should be explained based on direct evidence of non-fucosylated antibody action in human blood. Understanding the behavioral differences between non-fucosylated and fucosylated therapeutic antibodies in human blood can provide a new clue for improving antibody therapies.

In this study, we explored the mechanisms by which non-fucosylated therapeutic antibodies induce much higher ADCC in human blood than do fucosylated therapeutics. As a model of human *in vivo*, we employed a human B-cell depletion assay with anti-CD20 IgG1 rituximab, and focused on the actual binding to the two key cellular components of effector and target cells in human blood: namely, the binding through FcγRIIIa to natural killer (NK) cells (effector side interaction) and the binding through the antigen to target cells (target side interaction). This *ex vivo *model was considered to be suitable for analyzing the functions of therapeutic antibodies in human blood because it has been shown to reflect well the *in vivo *intravascular environment [18,20,22,23]. Here, we discuss two ingenious mechanisms underlying the enhanced ADCC efficacy of fully non-fucosylated therapeutic antibodies in human blood, based on data reflecting the actions of antibody molecules in human blood.

## Methods

### Blood donors

Blood donors were randomly selected from healthy volunteers registered at Tokyo Research Laboratories, Kyowa Hakko, Co., Ltd. All donors gave written informed consent before the analyses. Human serum samples collected from 12 individuals (5 women and 7 men, aged 20 to 57 years) with written informed consent were purchased from ProMedDx (Norton, MA).

### Antibodies

Non-fucosylated and fucosylated anti-CD20 IgG1s, and a triple amino acid-substituted anti-CD20 IgG1 rituximab mutant (S239D/S298A/I332E) were prepared as described previously [20,24]. Anti-CD20 IgG1s were biotinylated using EZ-Link Sulfo-NHS-LC-biotin (Pierce, Rock-ford, IL) according to the manufacturer's instructions. The labeled anti-CD20s were purified by dialysis with Spectra/Pro^® ^2 Dialysis Membrane (Spectrum Laboratories, Inc., Rancho Dominguez, CA) in phosphate-buffered saline (PBS). The antigen-binding activity of the labeled anti-CD20s was measured by CD20-binding ELISA [25], and confirmed to be equivalent to that of the non-labeled anti-CD20 and rituximab (Rituxan) purchased from Genentech, Inc. (South San Francisco, CA) prior to use.

### Analyses of human endogenous IgG1

Human endogenous IgG was fractionated from human serum samples of 20 healthy donors using MabSelect (Amersham Biosciences, Piscataway, NJ). The bound fraction contained IgG1, 2 and 4, whereas the bulk of IgG3 passed through the column. The pool of IgG1, 2, and 4, eluted from the MabSelect, was stored in 10 mM citrate buffer pH 6.0 with 0.15 M NaCl. The concentration of endogenous IgG1 in the pool was measured by human IgG1-specific ELISA using peroxidase-conjugated mouse anti-human IgG1 monoclonal antibodies (clone HP6069; Zymed Laboratories, South San Francisco, CA) as previously described [26]. The Fc of the IgG1 was prepared from the pool by a common procedure including papain cleavage, purification with MabSelect, and Superose12 (Amersham Biosciences) gel filtration chromatography to analyze the Fc oligosaccharide structure.

### Analyses of antibody-derived *N*-linked oligosaccharides

*N*-linked oligosaccharides were released by digestion of the purified anti-CD20 IgG1 and the Fc of human endogenous IgG1 with *N*-glycosidase F (Takara, Shiga, Japan). The released carbohydrates were analyzed by matrix-assisted laser desorption/ionization time-of-flight mass spectrometry (MALDI-TOF MS) with a positive-ion mode as described previously [27]. Monosaccharide composition of IgG1 was characterized by modified high-performance anion exchange chromatography (HPAEC) analysis as previously described [28].

### Antigen binding on target cells

Human peripheral blood mononuclear cells (PBMC) were prepared from healthy donors, and washed and resuspended in stain buffer (PBS containing 1% bovine serum albumin (BSA)). B cells were purified from PBMC by B Cell Isolation Kit II (Miltenyi Biotec, Bergisch-Gladbach, Germany) as negative fractions passed through a magnetic separation column (autoMACS™; Miltenyi Biotec). The purity of isolated B cells was more than 95%, as assessed by flow cytometer FACSCalibur (BD Biosciences, San Jose, CA) using anti-CD19-FITC (clone J4.119; Beckman Coulter, Coulter, Miami, FL). Purified B cells were adjusted to 1 × 10^6 ^cells/mL and incubated with serial dilutions (0.001 to 10 μg/mL) of anti-CD20 s for 30 min to 4 h at 37°C. The cells were then washed, and incubated with phycoerythrin (PE)-conjugated mouse anti-human IgG (BD Biosciences Pharmingen, San Diego, CA) for 30 min at 4°C to detect binding of anti-CD20s on B cells using FACSCalibur.

### *Ex vivo *B-cell depletion assay

Heparinized peripheral blood samples from healthy donors were incubated with serial dilutions (0.0001 to 10 μg/mL) of anti-CD20s for 4 h at 37°C, and CD19^+^CD2^- ^B cells were analyzed by FACSCalibur as described previously [20]. The cytotoxicity against CD19^+^CD2^- ^B cells was calculated according to the following formula:

Cytotoxicity (%) = (1 - (%CD19^+^CD2^- ^B cells treated with anti-CD20 IgG1)/(%CD19^+^CD2^- ^B cells treated with PBS)) × 100.

### Antigen-binding analysis in human blood

The antigen-binding activity of anti-CD20s on human B cells in human whole blood was analyzed by competitive inhibition assay with biotin-labeled anti-CD20 using flow cytometry. Heparinized peripheral blood from healthy donors was incubated simultaneously with 3.3 μg/mL of the biotinylated anti-CD20 (fucosylated or non-fucosylated) and serial dilutions (1 to 100 μg/mL) of non-labeled competitor anti-CD20s on ice for 30 min. Aliquots of samples were washed once with the stain buffer, and incubated with PE-conjugated streptavidin (Beckman Coulter) and anti-CD19-FITC on ice for 30 min. Stained cells were suspended in FACS Lysing Solution (BD Biosciences) to lyse erythrocytes and washed twice with the stain buffer. The binding intensity of biotinylated anti-CD20 to B cells detected with CD19^+ ^was analyzed by FACSCalibur.

### FcγRIIIa-binding analysis in human blood

The FcγRIIIa-binding activity of anti-CD20s on human NK cells in human whole blood was analyzed by flow cytometry using anti-idiotype monoclonal antibodies against rituximab [29]. Heparinized peripheral blood samples from healthy donors or samples, depleted of plasma by washing and reconstituted with stain buffer, were incubated with serial dilutions (1.1 to 30 μg/mL) of anti-CD20s on ice for 30 min. After washing with the stain buffer, the cells were stained with anti-rituximab-FITC (clone MB2 A4, Abcam, Cambridge, UK) to detect anti-CD20s bound to FcγR, and simultaneously stained with PE-Cyanin 5.1 (PC5)-labeled anti-CD3 antibodies PC5 (clone UCHT1, Beckman Coulter) and PE-labeled anti-CD56 antibodies (clone N901, Beckman Coulter) to detect CD3^-^CD56^+ ^NK cells. Following incubation on ice for 30 min, the intensity of anti-rituximab-FITC bound to NK cells detected with CD3^-^CD56^+ ^was analyzed by FACSCalibur. To analyze whether or not the binding of anti-CD20 to NK cells was through FcγRIIIa, samples were incubated simultaneously with 100 μg/mL of non-labeled anti-CD16 antibodies (clone 3G8, Ancell, Bayport, MN) and 10 μg/mL of non-fucosylated anti-CD20 on ice for 30 min, and the anti-CD20 bound to NK cells was analyzed by FACSCalibur. Mouse IgG1 (clone 15H6s, Beckman Coulter) was used as an isotype control.

### *In vitro *ADCC assay

Human PBMC, including effector NK cells and target B cells, was prepared from healthy donors and suspended in RPMI 1640 medium supplemented with 5% fetal bovine serum. Then, the PBMC suspension (240 μL including 0.6 × 10^6 ^cells), auto plasma or commercial human serum (300 μL), and serial dilutions (0.001 to 10 μg/mL) of anti-CD20s (60 μL in PBS) were dispensed into each well of 24-well culture plates and incubated at 37°C for 4 h. The cytotoxicity against CD19^+^CD2^- ^B cells was estimated as described above in an *ex vivo *B-cell depletion assay. The ADCC inhibitory activity of human serum was calculated according to the following formula:

ADCC inhibition (%) = (1 - (cytotoxicity (%) with human serum)/(cytotoxicity (%) without human serum)) × 100.

## Results

### Generation of non-fucosylated and fucosylated anti-CD20s

**Figure 1:**
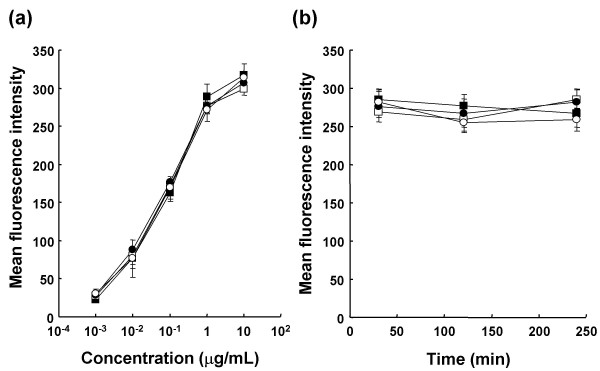
**Antigen binding activity of anti-CD20 IgG1 rituximab variants to target B cells**. Purified human B cells from healthy donor 1 were incubated with anti-CD20 IgG1 rituximab (open square) and its variants (fucosylated (closed circle), non-fucosylated (open circle), triple amino acid-substituted mutant S239D/S298A/I332E (closed square)) at indicated concentrations at 37°C for 30 min (a), or at 3.3 μg/mL at 37°C for 30, 120 and 240 minutes (b). In both experiments, anti-CD20s bound to CD20 on B cells were detected with PE-conjugated mouse anti-human IgG. Mean fluorescence intensity is indicated by mean values ± SD of triplicates.

**Figure 2:**
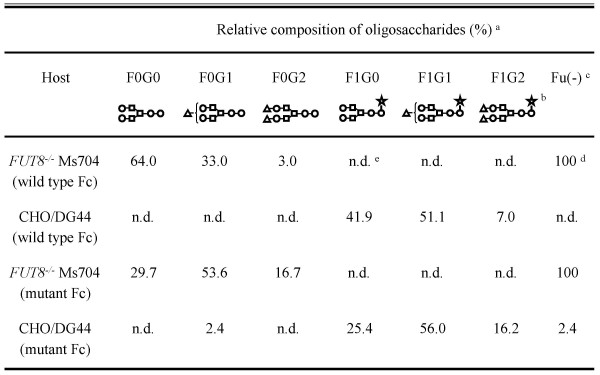
**Oligosaccharide analysis of the anti-CD20 IgG1 rituximab variants**. ^a ^Each value of composition is the relative amount in total complex-type oligosaccharides detected. ^b ^Schematic descriptions of oligosaccharides correspond to: galactose (triangle), *N*-acetylglucosamine (GlcNAc) (circle), mannose (square), and core fucose (star). ^c ^Relative amount of non-fucosylated oligosaccharides. ^d ^Data from reference 28. ^e ^n.d.: not detected (less than 2.0%).

Non-fucosylated and fucosylated anti-CD20s are composed of amino acid sequences identical to that of rituximab, and thus it was confirmed that they exhibited equal CD20 binding activity using the antigen-binding ELISA [20]. The anti-CD20 variant (S239D/S298A/I332E) also bound to the CD20 antigen equally as well as rituximab because the variable region of both forms had the same amino acid sequence [24]. Thus, these anti-CD20s also showed equal binding to human B cells in flow cytometry analyses, and down-modulation of CD20 by anti-CD20s was not observed during 4h-incubation (Fig. 1). Oligosaccharide analysis of the generated anti-CD20s confirmed that the attached oligosaccharides were of a complex biantennary type and that the IgG1 generated by the *FUT8*^-/- ^cell line Ms704 contained no fucose residue (Fig. 2).

### *Ex vivo *ADCC activity of anti-CD20s

**Figure 3:**
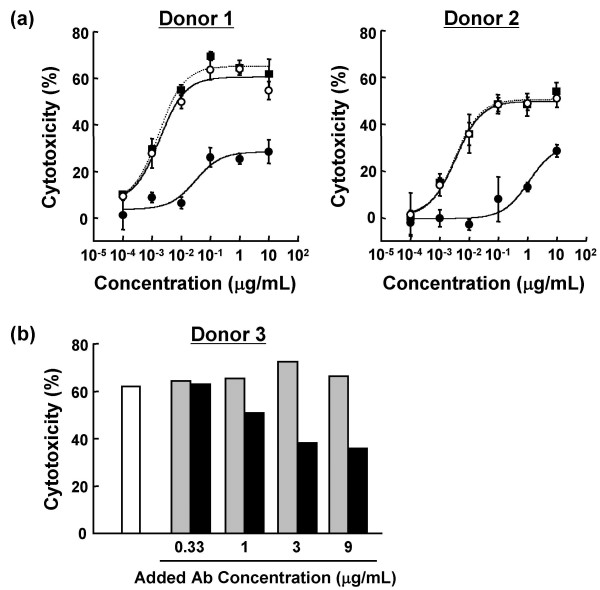
***Ex vivo *B-cell depletion activity of anti-CD20 IgG1 rituximab variants in a whole blood matrix**. (a) Heparinized peripheral blood from healthy donors 1 and 2 were incubated with serial dilutions of anti-CD20 IgG1 rituximab variants (fucosylated (closed circle), non-fucosylated (open circle), triple amino acid-substituted mutant S239D/S298A/I332E (closed square)). No significant difference in cytotoxicity was observed between non-fucosylated and triple amino acid-substituted mutant S239D/S298A/I332E by paired t-test. (b) Non-fucosylated anti-CD20 was added to the whole blood sample from donor 3 at 0.11 μg/mL (white column). Non-fucosylated (gray column) or fucosylated (black column) anti-CD20 was further added to the reaction at final concentrations as indicated. In both experiments, the remaining B cells after 4-h treatment were stained with anti-CD19-FITC, and the samples were analyzed on a flow cytometer to quantify the number of B cells. B-cell depletion is represented as cytotoxicity (%) as described in Materials and Methods.

To analyze the *ex vivo *ADCC activity of each anti-CD20, whole blood samples from two healthy donors (donors 1 and 2) were incubated with serial dilutions of non-fucosylated or fucosylated anti-CD20s (Fig. 3a). Fucosylated anti-CD20 reduced only about 30% of B cells at 10 μg/mL in donors 1 and 2. By contrast, non-fucosylated anti-CD20 showed more than 100-fold potent B-cell depletion activity with much higher efficacy than its fucosylated counterpart in both individuals, with EC_50 _values of 0.001 μg/mL (in donor 1) and 0.01 μg/mL (in donor 2). The anti-CD20 variant (S239D/S298A/I332E), having higher FcγRIIIa-binding activity than fucosylated wild type anti-CD20, also showed high *ex vivo *ADCC almost identical to that of non-fucosylated wild type anti-CD20 in both individuals, consistent with the findings of previous reports [21,24]. A series of mixtures composed of non-fucosylated and fucosylated anti-CD20s at different ratios was prepared by adding serial 3-fold amounts of fucosylated anti-CD20 to a fixed amount (0.11 μg/mL) of non-fucosylated anti-CD20, and *ex vivo *B-cell depletion activities of the mixtures were measured (Fig. 3b, donor 3). The concentration of 0.11 μg/mL was chosen to be the minimum dose showing saturable *ex vivo *efficacy in the donor blood (donor 3). There was no significant change observed by the addition of further non-fucosylated anti-CD20, as the initial dose already reached the saturable effective dose. The addition of the fucosylated anti-CD20 to non-fucosylated anti-CD20 depressed the *ex vivo *B-cell depletion activity in a dose-dependent manner; thus, the *ex vivo *B-cell depletion activity of the mixture consisting of fucosylated and non-fucosylated anti-CD20s did not reach that of the antibody sample consisting of non-fucosylated anti-CD20 alone.

### Inhibitory effect of human plasma on binding of anti-CD20s to FcγRIIIa on NK cells

**Figure 4:**
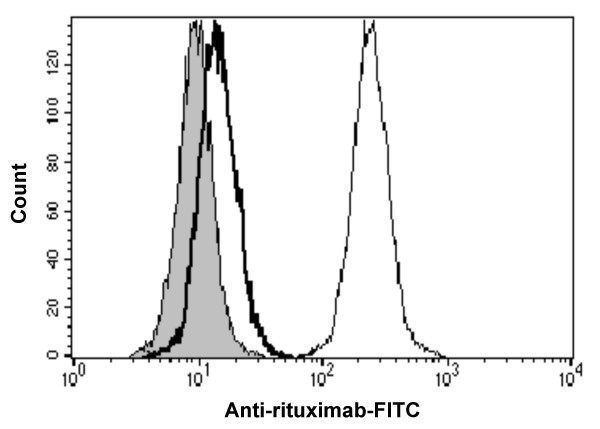
***Ex vivo *binding of non-fucosylated anti-CD20 IgG1 rituximab to FcγRIIIa on NK cells**. Heparinized peripheral blood from donor 4 was incubated with non-fucosylated anti-CD20 IgG1 10 μg/mL (blank histogram) or stain buffer alone (filled histogram), in the presence of anti-CD16 antibody 3G8 (bold line) or mouse IgG1 isotype control (solid line). CD3^-^CD56^+ ^NK cells were gated in the histogram. The binding of non-fucosylated anti-CD20 IgG1 to FcγRIIIa on NK cells was detected by anti-rituximab-FITC.

**Figure 5:**
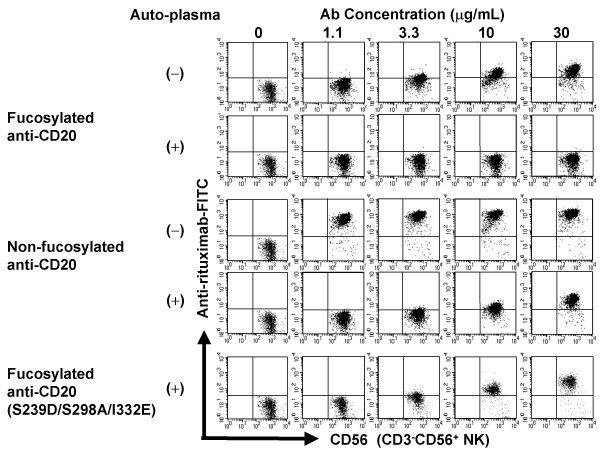
***Ex vivo *binding activity of anti-CD20 IgG1 rituximab variants to FcγRIIIa on NK cells**. Heparinized peripheral blood from donor 4, or blood depleted of auto-plasma by washing and reconstitution with stain buffer, was incubated with serial dilutions of anti-CD20 IgG1s. Anti-CD20 IgG1 rituximab variants bound to FcγRIIIa on CD3^-^CD56^+ ^NK cells after incubation on ice for 30 min were detected by anti-rituximab-FITC.

To address the reason why non-fucosylated anti-CD20 induced a higher *ex vivo *ADCC efficacy than did fucosylated anti-CD20, the binding of anti-CD20s to NK cells was analyzed in human whole blood matrices using anti-rituximab-FITC in donor 4, who showed a similar B-cell depletion pattern with anti-CD20 as donor 2. The binding of anti-CD20 to NK cells was confirmed to be mediated mostly through the interaction of the Fc region with FcγRIIIa on NK cells since anti-CD16 antibody 3G8 inhibited the binding (Fig. 4). Non-fucosylated anti-CD20 exhibited markedly stronger binding to NK cells than fucosylated anti-CD20 in terms of the ratio of anti-CD20 positive NK cells and the staining intensity of anti-CD20 per NK cell (Fig. 5). It is worth noting that non-fucosylated and variant (S239D/S298A/I332E) anti-CD20s still retained high binding activity for NK cells in the presence of plasma, while the binding of fucosylated anti-CD20 to NK cells was almost abolished in the presence of plasma.

### Inhibitory effect of fucosylated anti-CD20 on binding of non-fucosylated anti-CD20 to the target antigen on B cells

**Figure 6:**
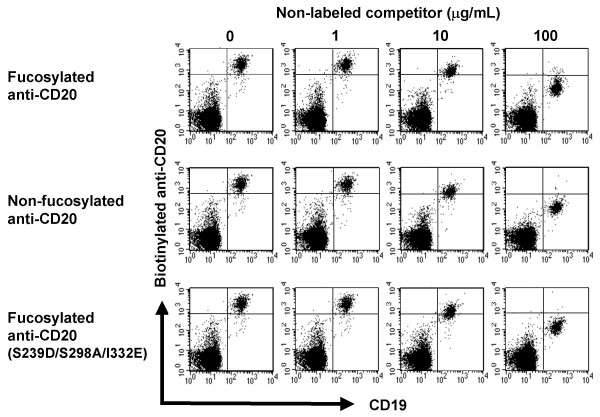
***Ex vivo *binding activity of anti-CD20 IgG1 rituximab variants to CD20 on B cells**. Heparinized peripheral blood from donor 5 was incubated simultaneously with 3.3 μg/mL of biotinylated anti-CD20 IgG1 and serial dilutions of non-labeled competitor anti-CD20 IgG1 rituximab on ice for 30 min. The remaining biotinylated anti-CD20 IgG1 bound to CD20^+ ^B cell was detected by streptavidin-PE using FACS analysis. Data for the binding of biotinylated fucosylated anti-CD20 are shown.

The binding of anti-CD20 to target B cells was also analyzed in human whole blood matrices (donor 5) using a competitive inhibition assay with biotin-labeled anti-CD20s. Biotinylated anti-CD20s that retained antigen-binding activity comparable to that of non-labeled anti-CD20 were employed in the competitive inhibition assay. As shown in Fig. 6, the *ex vivo *binding of biotinylated anti-CD20 (3.3 μg/mL) was inhibited by non-labeled anti-CD20 in a dose-dependent manner, irrespective of fucosylation or amino acid mutation of the Fc region of the added anti-CD20; i.e., fucosylated, non-fucosylated and triple amino-acid-substituted anti-CD20s exhibited an identical antigen-binding activity on the target B cells in human whole blood.

### Inhibitory effect of individual human plasma on *in vitro *ADCC induced by anti-CD20s

**Figure 7:**
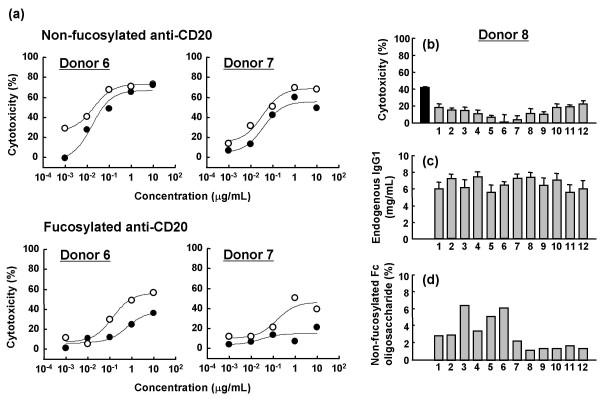
**Influence of human plasma on ADCC activity of fucosylated anti-CD20**. (a) B-cell depletion activity of non-fucosylated and fucosylated anti-CD20 was analyzed using purified PBMC with (closed circle) or without (open circle) auto-plasma (donors 6 and 7). Cytotoxicity (%) after 4-h treatment was calculated as described in Materials and Methods. (b) B-cell depletion activity of fucosylated anti-CD20 was analyzed using purified PBMC from donor 8 with (gray column) or without (black column) allo-serum from 12 healthy adult individuals. Cytotoxicity (%) after 4-h treatment was calculated as described in Materials and Methods, and is indicated by mean values ± SD of triplicates. Individual differences in endogenous IgG1 profiles of the allo-serum, endogenous IgG1 concentration (c) (indicated by mean values ± SD of triplicates) and relative amounts of non-fucosylated IgG1 Fc oligosaccharide (d) were also analyzed. The fucosylated and non-fucosylated anti-CD20 IgG1s (Fig. 2) with known fucosylation level and antibody concentration (2 mg/mL and 10 mg/mL) were used as standard antibodies for experiments (c) and (d).

The inhibitory effect of human plasma on ADCC induced by anti-CD20s was confirmed by an *in vitro *ADCC assay with purified PBMC in the presence or absence of plasma (donors 6 and 7). In this assay, fucosylated or non-fucosylated anti-CD20 (1 μg/mL) was employed as an ADCC inducer against target B cells, and B-cell depletion mediated by the ADCC was measured by flow cytometry. The ADCC of anti-CD20s was inhibited by autologous plasma (greatly in fucosylated and slightly in non-fucosylated anti-CD20s) (Fig. 7a). However, in the absence of plasma, fucosylated anti-CD20 showed quite high ADCC (exceeding 40% cytotoxicity) compared with the case in the presence of plasma, and this value was fairly close to that of non-fucosylated anti-CD20. Next, we focused on whether or not individual differences in endogenous human plasma modulate the inhibitory effects on ADCC induced by fucosylated anti-CD20 (1 μg/mL) (Fig. 7b, c, d, donor 8). Human sera from 12 healthy individuals were employed; the individual sera themselves were confirmed not to show any detectable cytotoxic activity against human B cells in this experiment. Although the 12 individual sera displayed slight differences in terms of IgG1 concentration (5.3 to 7.5 mg/mL) and the relative amount of non-fucosylated Fc oligosaccharides (1.2 to 6.5%), consistent with previous reports [30,31], the intensity of inhibitory activity of individual serum against the ADCC of fucosylated anti-CD20 did not significantly correlate with these differences.

## Discussion

Currently, the application of human IgG1 fully lacking core fucose in the Fc oligosaccharides is expected to be a promising approach for next-generation therapeutic antibodies with improved efficacy, even when administrated at low doses in humans *in vivo*. In order to develop fully non-fucosylated therapeutic antibodies as new medicine, the pharmacodynamic characteristics of the antibodies should be elucidated: how fully non-fucosylated antibodies effectively mediate two key cellular component interactions of effector and target cells to exercise high ADCC and what the differences are between non-fucosylated and fucosylated antibodies in these actions. In this study, we addressed non-fucosylated antibody actions in human blood.

Firstly, we were surprised that the binding of fucosylated antibodies to NK cells was not detected, even at the high concentration of 30 μg/mL, in the presence of human plasma (Fig. 5). A common feature of the antibody therapies against cancers is that their anti-tumor efficacies typically require weekly administration of a high dose (2–8 mg/kg) over several months to maintain effective serum concentrations exceeding 10 μg/mL [32-34]. Thus, it is realized that currently-licensed therapeutic antibodies mostly do not bind to NK cells in human blood after administration. On the other hand, the binding of antibodies to target cells was detected at a concentration of 3.3 μg/mL, irrespective of the presence of human plasma (Fig. 1, 6). The antigen binding of therapeutic antibodies to target cells appears to be simply determined by their specificity and affinity. In current antibody therapies, administered therapeutic antibodies seem to have sufficient capability to concentrate in the target cells accessible from the bloodstream, but not to capture effector cells in the target sites effectively.

We confirmed the inhibitory effects of human serum IgG on the ADCC of therapeutic antibodies, consistent with previous reports [18-21,35]. ADCC of fucosylated antibodies was significantly affected by human plasma IgG (Fig. 7a). In contrast, non-fucosylated antibodies showed much stronger binding to FcγRIIIa on NK cells, even in the presence of human plasma IgG, resulting in much higher potency and efficacy of non-fucosylated compared to fucosylated antibodies (Fig. 3a). In our experiments, the concentrations of therapeutic antibodies required for inducing *ex vivo *ADCC and binding to FcγRIIIa on NK cells were not necessarily consistent. This discrepancy seemed to reflect the fact that human serum IgG bound to FcγRIIIa on NK cells might be more effectively displaced by therapeutic antibodies bound to the surface of target cells than unbound forms, possibly due to an avidity effect via formation of a polyvalent FcγRIIIa-binding matrix, and consequently promoting recruitment of NK cells. Interestingly, a triple amino acid-substituted mutant (S239D/S298A/I332E) showed almost identical *ex vivo *ADCC and NK cell binding to that of the non-fucosylated wild type (Fig. 3a, 5), although the mutant was shown to have higher affinity than the non-fucosylated wild type for recombinant FcγRIIIa *in vitro *[24]. The binding affinity estimated by solid-phase assay with recombinant protein forms might not necessarily be the same as the affinity to the native structure forms on the cell surface; thus, the equivalency in anti-CD20 efficacy was observed.

Before starting these experiments, we speculated that the concentration of human plasma IgG1 is so huge that its fucosylation level might affect the inhibitory activity of ADCC induced by therapeutic antibodies, even though the proportion of non-fucosylated IgG1 is very low. Indeed, human plasma contains 5 mg/mL of endogenous IgG1 [30], which is approximately 100-fold higher than the serum trough concentration of rituximab [32]. However, contrary to our speculation, we could not detect any significant difference caused by variations in serum IgG1 fucosylation levels in healthy individuals (Fig. 7) although individual human serum IgG1 from 12 healthy donors showed slight variations in the concentration (5.3 to 7.5 mg/mL) and the content of non-fucosylated oligosaccharide structures (1.2 to 6.5%). Factors other than endogenous IgG1 concentration and fucosylation levels, such as variations of other endogenous IgG subclasses, might also be involved in the ADCC inhibitory activity of human serum IgG from healthy individuals. Some leukemia patients are reported to increase their relative levels of non-fucosylated Fc oligosaccharides [36]. It is still thought to be interesting to explore whether individual patient variations in endogenous IgG1 fucosylation level might affect the efficacy of antibody therapies.

We also confirmed that enhanced *ex vivo *ADCC of non-fucosylated therapeutic antibodies is inhibited by their fucosylated counterparts, consistent with previous reports [20,21,27]. In our experiments, down-modulation of CD20 antigen on B cells mediated by anti-CD20 was not observed (Fig. 1), and simple competition for binding to the antigens on target B cells between fucosylated and non-fucosylated anti-CD20s was detected in human blood (Fig. 6), resulting in inhibition of the enhanced ADCC of non-fucosylated anti-CD20 by fucosylated anti-CD20. Thus, the mechanism by which ADCC induced by non-fucosylated antibodies is inhibited by fucosylated forms is mainly through competition of the two forms for the antigens on target cells. In other words, the density of the non-fucosylated form on the target cells is reduced by fucosylated form occupation, which causes an effect to shed the target antigens from capture by the therapeutic agent having high ADCC activity even in plasma. To overcome interference from human plasma IgG and recruit NK cells to the target cells effectively, the key is to coat the target cells with therapeutic agents having higher binding affinity to FcγRIIIa than plasma IgG, such as fully non-fucosylated IgG1. The non-fucosylated form of IgG1 is a natural component in human serum IgG, and is also observed as a trace ingredient in currently-licensed therapeutic antibodies [37-40]. It will be great benefits for patients if the proportion of the non-fucosylated form is increased in antibody therapeutics.

## Conclusion

Our findings demonstrate that removal of fucosylated antibody ingredients from antibody therapeutics elicits high ADCC, even in human blood, via two mechanisms: namely, by evading the inhibitory effects both of plasma IgG on the binding of FcγRIIIa on NK cells and of fucosylated counterparts on the binding of the antigen on target cells. Thus, antibody therapy consisting of fully non-fucosylated IgG1, not including the fucosylated form, is worth to apply as next-generation antibody therapy with improved efficacy at a reduced dose for patients, which will have a great impact especially on human cancer therapy based on the ADCC mechanism.

## Competing interests

The authors declare that they have no competing interests.

## Authors' contributions

SI designed the study; participated in the purification and the biological activity analysis of the antibodies; carried out the *ex vivo *and *in vitro *ADCC assays; and drafted the manuscript. RKK participated in carrying out the flow cytometric analysis. KM participated in the FcγRIIIa binding analysis of the antibodies. HM participated in carrying out the *ex vivo *and *in vitro *ADCC assays. MI participated in the purification and biological analysis of human endogenous IgG1. AO participated in the analysis of antibody-derived *N*-linked Fc oligosaccharides. KS was the director of the laboratories that carried out the present study and also discussed the study from scientific points of view. MS was the head of the laboratories that carried out the present study, initially conceived of the study, and helped to design the study and drafted the manuscript. All authors read and approved the final manuscript.

## Pre-publication history

The pre-publication history for this paper can be accessed here:

http://www.biomedcentral.com/1471-2407/9/58/prepub

## References

[B1] de Bono JS Rowinsky EK The ErbB receptor family: a therapeutic target for cancer Trends Mol Med 2002 8 Suppl 4 S19 26 10.1016/S1471-4914(02)02306-7 11927283

[B2] Forero A Lobuglio AF History of antibody therapy for non-Hodgkin's lymphoma Semin Oncol 2003 30 1 5 10.1053/j.seminoncol.2003.10.002 14710396

[B3] Grillo-Lopez AJ Rituximab (Rituxan/Mab Thera): the first decade (1993–2003) Expert Rev Anticancer Ther 2003 3 767 769 10.1586/14737140.3.6.767 14686699

[B4] Vogel CL Franco SX Clinical experience with trastuzumab (Herceptin) Breast J 2003 9 452 462 10.1046/j.1524-4741.2003.09602.x 14616939

[B5] Reichert JM Rosensweig CJ Faden LB Dewitz MC Monoclonal antibody successes in the clinic Nat Biotechnol 2005 23 1073 1078 10.1038/nbt0905-1073 16151394

[B6] Shan D Ledbetter JA Press OW Signaling events involved in anti-CD20-induced apoptosis of malignant human B cells Cancer Immunol Immunother 2000 48 673 683 10.1007/s002620050016 10752475 PMC11037214

[B7] Carter P Improving the efficacy of antibody-based cancer therapies Nat Rev Cancer 2001 1 118 129 10.1038/35101072 11905803

[B8] Glennie MJ Winkel JG van de Renaissance of cancer therapeutic antibodies Drug Discov Today 2003 8 503 510 10.1016/S1359-6446(03)02714-4 12818520

[B9] Smith MR Rituximab (monoclonal anti-CD20 antibody): mechanisms of action and resistance Oncogene 2003 22 7359 7368 10.1038/sj.onc.1206939 14576843

[B10] Cartron G Dacheux L Salles G Solal-Celigny P Bardos P Colombat P Watier H Therapeutic activity of humanized anti-CD20 monoclonal antibody and polymorphism in IgG Fc receptor FcγRIIIa gene Blood 2002 99 754 758 10.1182/blood.V99.3.754 11806974

[B11] Anolik JH Campbell D Felgar RE Young F Sanz I Rosenblatt J Looney RJ The relationship of FcγRIIIa genotype to degree of B cell depletion by rituximab in the treatment of systemic lupus erythematosus Arthritis Rheum 2003 48 455 459 10.1002/art.10764 12571855

[B12] Weng WK Levy R Two immunoglobulin G fragment C receptor polymorphisms independently predict response to rituximab in patients with follicular lymphoma J Clin Oncol 2003 21 3940 3947 10.1200/JCO.2003.05.013 12975461

[B13] Dall'Ozzo S Tartas S Paintaud G Cartron G Colombat P Bardos P Watier H Thibault G Rituximab-dependent cytotoxicity by natural killer cells: influence of FCGR3A polymorphism on the concentration-effect relationship Cancer Res 2004 64 4664 4669 10.1158/0008-5472.CAN-03-2862 15231679

[B14] Gennari R Menard S Fagnoni F Ponchio L Scelsi M Tagliabue E Castiglioni F Villani L Magalotti C Gibelli N Oliviero B Ballardini B Da Prada G Zambelli A Costa A Pilot study of the mechanism of action of preoperative trastuzumab in patients with primary operable breast tumors overexpressing HER2 Clin Cancer Res 2004 10 5650 5655 10.1158/1078-0432.CCR-04-0225 15355889

[B15] Louis E El Ghoul Z Vermeire S Dall'Ozzo S Rutgeerts P Paintaud G Belaiche J De Vos M Van Gossum A Colombel JF Watier H Association between polymorphism in IgG Fc receptor IIIa coding gene and biological response to infliximab in Crohn's disease Aliment Pharmacol Ther 2004 19 511 519 10.1111/j.1365-2036.2004.01871.x 14987319

[B16] Miescher S Spycher MO Amstutz H De Haas M Kleijer M Kalus UJ Radtke H Hubsch A Andresen I Martin RM Bichler J A single recombinant anti-RhD IgG prevents RhD immunization: association of RhD-positive red blood cell clearance rate with polymorphisms in the FcγRIIA and FcγIIIA genes Blood 2004 103 4028 4035 10.1182/blood-2003-11-3929 14976055

[B17] Treon SP Hansen M Branagan AR Verselis S Emmanouilides C Kimby E Frankel SR Touroutoglou N Turnbull B Anderson KC Maloney DG Fox EA Polymorphisms in FcγRIIIA (CD16) receptor expression are associated with clinical response to rituximab in Waldenström's macroglobulinemia J Clin Oncol 2005 23 474 481 10.1200/JCO.2005.06.059 15659493

[B18] Vugmeyster Y Howell K Rituximab-mediated depletion of cynomolgus monkey B cells in vitro in different matrices: possible inhibitory effect of IgG Int Immunopharmacol 2004 4 1117 1124 10.1016/j.intimp.2004.04.015 15222987

[B19] Preithner S Elm S Lippold S Locher M Wolf A da Silva AJ Baeuerle PA Prang NS High concentrations of therapeutic IgG1 antibodies are needed to compensate for inhibition of antibody-dependent cellular cytotoxicity by excess endogenous immunoglobulin G Mol Immunol 2006 43 1183 1193 10.1016/j.molimm.2005.07.010 16102830

[B20] Iida S Misaka H Inoue M Shibata M Nakano R Yamane-Ohnuki N Wakitani M Yano K Shitara K Satoh M Nonfucosylated therapeutic IgG1 antibody can evade the inhibitory effect of serum immunoglobulin G on antibody-dependent cellular cytotoxicity through its high binding to FcγRIIIa Clin Cancer Res 2006 12 2879 2887 10.1158/1078-0432.CCR-05-2619 16675584

[B21] Satoh M Iida S Shitara K Non-fucosylated therapeutic antibodies as next-generation therapeutic antibodies Expert Opin Biol Ther 2006 6 1161 1173 10.1517/14712598.6.11.1161 17049014

[B22] Vugmeyster Y Howell K Bakshl A Flores C Canova-Davis E Effect of anti-CD20 monoclonal antibody, Rituxan, on cynomolgus monkey and human B cells in a whole blood matrix Cytometry A 2003 52 101 109 10.1002/cyto.a.10030 12655653

[B23] Lazar GA Dang W Karki S Vafa O Peng JS Hyun L Chan C Chung HS Eivazi A Yoder SC Vielmetter J Carmichael DF Hayes RJ Dahiyat BI Engineered antibody Fc variants with enhanced effector function Proc Natl Acad Sci USA 2006 103 4005 4010 16537476 10.1073/pnas.0508123103 PMC1389705

[B24] Masuda K Kubota T Kaneko E Iida S Wakitani M Kobayashi-Natsume Y Kubota A Shitara K Nakamura K Enhanced binding affinity for FcγRIIIa of fucose-negative antibody is sufficient to induce maximal antibody-dependent cellular cytotoxicity Mol Immunol 2007 44 3122 3131 10.1016/j.molimm.2007.02.005 17379311

[B25] Yamane-Ohnuki N Kinoshita S Inoue-Urakubo M Kusunoki M Iida S Nakano R Wakitani M Niwa R Sakurada M Uchida K Shitara K Satoh M Establishment of *FUT8 *knockout Chinese hamster ovary cells: an ideal host cell line for producing completely defucosylated antibodies with enhanced antibody-dependent cellular cytotoxicity Biotechnol Bioeng 2004 87 614 622 10.1002/bit.20151 15352059

[B26] Jefferis R Reimer CB Skvaril F de Lange GG Goodall DM Bentley TL Phillips DJ Vlug A Harada S Radl J Claassen E Boersma JA Coolen J Evaluation of monoclonal antibodies having specificity for human IgG subclasses: results of the 2nd IUIS/WHO collaborative study Immunol Lett 1992 31 143 168 10.1016/0165-2478(92)90141-A 1371266

[B27] Kanda Y Yamada T Mori K Okazaki A Inoue M Kitajima-Miyama K Kuni-Kamochi R Nakano R Yano K Kakita S Shitara K Satoh M Comparison of biological activity among nonfucosylated therapeutic IgG1 antibodies with three different *N*-linked Fc oligosaccharides: the high-mannose, hybrid, and complex types Glycobiology 2007 17 104 118 10.1093/glycob/cwl057 17012310

[B28] Shinkawa T Nakamura K Yamane N Shoji-Hosaka E Kanda Y Sakurada M Uchida K Anazawa H Satoh M Yamasaki M Hanai N Shitara K The absence of fucose but not the presence of galactose or bisecting *N*-acetylglucosamine of human IgG1 complex-type oligosaccharides shows the critical role of enhancing antibody-dependent cellular cytotoxicity J Biol Chem 2003 278 3466 3473 10.1074/jbc.M210665200 12427744

[B29] Cragg MS Bayne MB Tutt AL French RR Beers S Glennie MJ Illidge TM A new anti-idiotype antibody capable of binding rituximab on the surface of lymphoma cells Blood 2004 104 2540 2542 10.1182/blood-2004-05-1733 15213098

[B30] Schauer U Stemberg F Rieger CH Borte M Schubert S Riedel F Herz U Renz H Wick M Carr-Smith HD Bradwell AR Herzog W IgG subclass concentrations in certified reference material 470 and reference values for children and adults determined with the binding site reagents Clin Chem 2003 49 1924 1929 10.1373/clinchem.2003.022350 14578325

[B31] Kuroda Y Nakata M Makino A Matsumoto A Ohashi K Itahashi K Takeuchi F Goto M Kojima N Mizuochi T Structural studies on IgG oligosaccharides of patients with primary Sjogren's syndrome Glycoconj J 2002 19 23 31 10.1023/A:1022528829799 12652077

[B32] Berinstein NL Grillo-López AJ White CA Bence-Bruckler I Maloney D Czuczman M Green D Rosenberg J McLaughlin P Shen D Association of serum rituximab (IDEC-C2B8) concentration and anti-tumor response in the treatment of recurrent low-grade or follicular non-Hodgkin's lymphoma Ann Oncol 1998 9 995 1001 10.1023/A:1008416911099 9818074

[B33] Goldenberg MM Trastuzumab, a recombinant DNA-derived humanized monoclonal antibody, a novel agent for the treatment of metastatic breast cancer Clin Ther 1999 21 309 318 10.1016/S0149-2918(00)88288-0 10211534

[B34] Baselga J Albanell J Mechanism of action of anti-HER2 monoclonal antibodies Ann Oncol 2001 12 Suppl 1 S35 41 10.1023/A:1011163824080 11521720

[B35] Nechansky A Schuster M Jost W Siegl P Wiederkum S Gorr G Kircheis R Compensation of endogenous IgG mediated inhibition of antibody-dependent cellular cytotoxicity by glyco-engineering of therapeutic antibodies Mol Immunol 2007 44 1815 1817 10.1016/j.molimm.2006.08.013 17011625

[B36] Kondo A Hosokawa Y Kiso M Hasegawa A Kato I Analysis of oligosaccharides of human IgG from serum of leukemia patients Biochem Mol Biol Int 1994 32 897 902 8069239

[B37] Mizuochi T Taniguchi T Shimizu A Kobata A Structural and numerical variations of the carbohydrate moiety of immunoglobulin G J Immunol 1982 129 2016 2020 6811655

[B38] Harada H Kamei M Tokumoto Y Yui S Koyama F Kochibe N Endo T Kobata A Systematic fractionation of oligosaccharides of human immunoglobulin G by serial affinity chromatography on immobilized lectin columns Anal Biochem 1987 164 374 381 10.1016/0003-2697(87)90507-0 3674386

[B39] Schenerman MA Hope JN Kletke C Singh JK Kimura R Tsao EI Folena-Wasserman G Comparability testing of a humanized monoclonal antibody (Synagis) to support cell line stability, process validation, and scale-up for manufacturing Biologicals 1999 27 203 215 10.1006/biol.1999.0179 10652176

[B40] Kamoda S Nomura C Kinoshita M Nishiura S Ishikawa R Kakehi K Kawasaki N Hayakawa T Profiling analysis of oligosaccharides in antibody pharmaceuticals by capillary electrophoresis J Chromatogr A 2004 1050 211 216 15508314

